# Delayed efficacy of radiofrequency catheter ablation on arrhythmias originating in the interventricular basal septum

**DOI:** 10.1002/ccr3.1883

**Published:** 2019-01-09

**Authors:** Marco V. Mariani, Maria C. Gatto, Agostino Piro, Francesco Fedele, Carlo Lavalle

**Affiliations:** ^1^ Department of Cardiovascular, Respiratory, Nephrology, Anaesthesiology and Geriatric Sciences of “Sapienza” University of Rome Rome Italy

**Keywords:** arrhythmia‐induced cardiomyopathy, Idiopathic ventricular arrhythmias, intramural focus, premature ventricular contraction, radiofrequency delayed efficacy

## Abstract

Delayed efficacy of radiofrequency energy can suppress ventricular arrhythmias after a failed ablation procedure. The implant of cardiac defibrillator for arrhythmia‐induced cardiomyopathy should be procrastinated after a period of follow‐up. Waiting for delayed efficacy is a reasonable choice to reduce the risk of complications associated with aggressive ablative approaches.

## 1 INTRODUCTION

Idiopathic outflow tract ventricular arrhythmias (IOT‐VAs) are ventricular tachycardias (VTs) or premature ventricular contractions (PVCs) presumably not related to myocardial scar. Although the right ventricle outflow tract (RVOT) is the most common site of origin for OT‐VAs, these arrhythmias can less frequently originate from the left ventricle outflow tract (LVOT). In general, VAs focus is more often located in the endocardium but sometimes it has an epicardial or intramural position. Idiopathic VAs usually arise from an intramural site of origin. OT‐VAs are generally benign and may require treatment if they are symptomatic, incessant or give rise to cardiomyopathy. Radiofrequency catheter ablation (RFCA) is an effective and safe therapeutic strategy that usually leads to immediate suppression of OT‐VAs. Here, we present one case of arrhythmia‐induced cardiomyopathy (AIC) derived from intramural idiopathic VAs originating in the basal interventricular septum (IVS) with reversal of left ventricular (LV) dilatation and improved LV ejection fraction (EF) after the elimination of the arrhythmia by the delayed effect of RFCA.

## 2 CASE REPORT

A 56‐year‐old man suffering from dyspnea for mild efforts was admitted to our hospital. He denied home therapy and he had a history of systemic arterial hypertension, obesity, sleep apnea, and persistent atrial fibrillation treated with electrical cardioversion in 2015, without known recurrences.

His physical examination pointed out cardiac arrhythmic activity, pulmonary congestion, jugular venous distension, hepatomegaly, and mild ankles swelling. Blood tests in the emergency department suggested acute heart failure with mild elevation of myocardial necrosis indices (pro‐BNP 5264 pg/mL, LDH 256 UI/L, CPK 597 UI/L, troponin T hs 0.023 μg/L with normal value <0.014 μg/L). Hilar congestion and cardiomegaly resulted from chest x‐ray examination.

**Figure 1:**
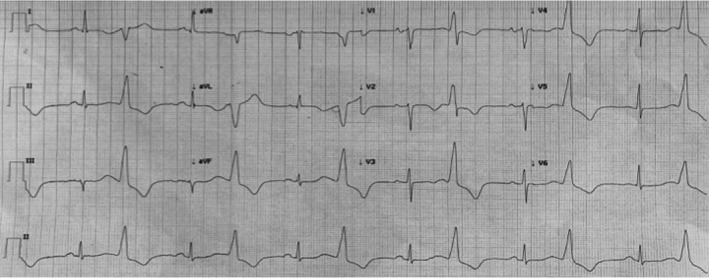
A 12‐lead electrocardiogram of the clinical arrhythmia. PVCs have RBBB and inferior QRS axis

The 12‐lead ECG showed sinus rhythm with frequent monomorphic PVCs in bigeminy pattern, short runs of nonsustained VT (NSVT), and accelerated idioventricular rhythm. PVCs had right bundle branch block (RBBB) pattern, precordial transition in lead V1, and inferior QRS axis (Figure 1).

Transthoracic echocardiogram revealed severely reduced systolic\function (EF 23%) with global hypokinesia, mild left ventricular dilatation (LVEDD 60 mm), normal LV mass, and wall thicknesses. Moreover, moderate mitral regurgitation and restrictive transmitral flow pattern were detected.

The coronary angiography was performed and no coronary stenoses were found.

Heart failure therapy was started. Because of the limited effectiveness of beta‐blockade on PVCs and NSVT, amiodarone was administered, but it caused prolongation of QTc interval requiring suspension of the antiarrhythmic drug.

In the suspicion of acute viral myocarditis, the serology for the most common cardiotropic viruses was tested but resulted negative. Due to the impossibility to suppress arrhythmias and to perform real‐time sequences, cardiac magnetic resonance imaging (CMR) was not realized.

**Figure 2:**
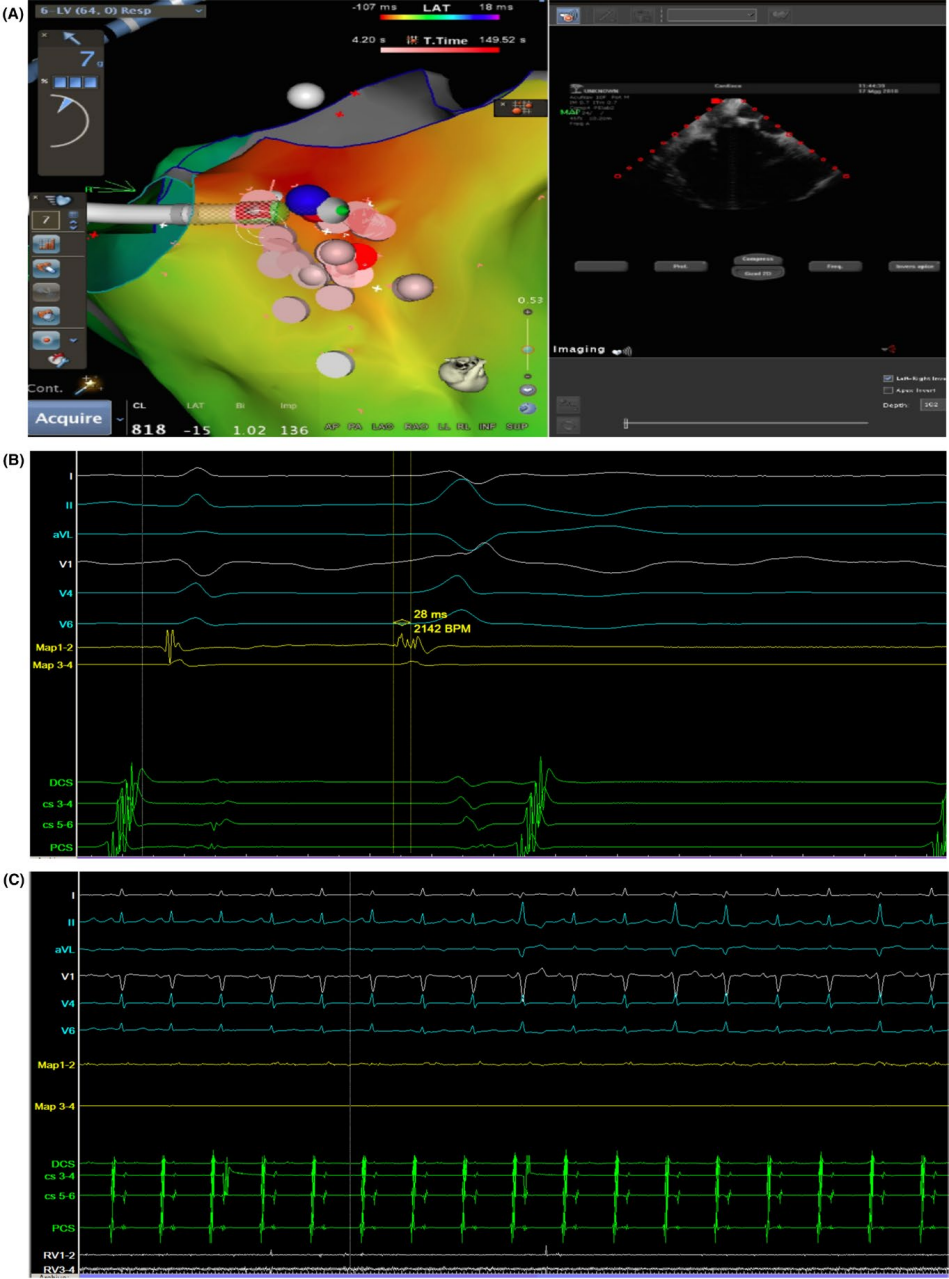
Mapping and ablation of the LV. Activation map of the LV guided by ICE (A). Surface lead recordings during PVC and recordings from a multipolar catheter located in proximity of the anterior part of the AMC (Map 1‐2 indicates distal electrode pair; Map 3‐4 indicates proximal electrode pair). The earliest site was recorded by the distal electrode pair Map 1‐2 preceding the onset of PVC by 28 ms (B). The suppression of PVCs during RF energy delivery (C)

The electrophysiological study was performed with electroanatomical mapping system (Carto® 3, Biosense Webster) guided by intracardiac echocardiography (ICE) (Figure 2A). The high‐density substrate mapping did not found any scar zones. Through the right femoral artery, an irrigated‐tip ablation catheter (ThermoCool® SmartTouch® Catheter, Biosense Webster Inc, Irvine, CA, US) was inserted via a retrograde transaortic fashion for mapping and ablation. The earliest activation site was found in close proximity to the anterior part of the aorto‐mitral continuity (AMC), which was prior to onset of QRS 28 ms (Figure 2B). Multiple RF applications were delivered to the earliest activation site with a target temperature of 43°C and a maximum power of 40 W, resulting in transient suppression of PVCs during erogations and early recurrence after the end of deliveries (Figure 2C). Electroanatomical mapping of RVOT was also performed in order to exclude a right exit of VAs, with multiple RF deliveries at the earliest activation site in the postero‐septal RVOT wall which showed a local presystolic activation of 25 ms. As in LVOT ablation attempts, RF resulted in rapid disappearance and early recurrence of PVCs. A nonmatching pace‐map resulted when pacing from the earliest site of endocardial activation at both sides of the septum. These features suggested an intramural focus within the interventricular basal septum. Mapping the site of origin via venous system was not accomplished because the mapping catheter could not be positioned in a perforator branch of the great cardiac vein (GCV). Hence, after many RF energy deliveries, the procedure was aborted, resulting in failure to suppress PVCs.

After 72 hours of observation, PVCs disappeared although there were no changes in medical therapy. One week after the PVCs disappearance, transthoracic echocardiogram showed improved systolic performance with a LVEF of 40% and slight reduction in LV diameter. After one month of follow‐up, cardiac function was completely normalized and ambulatory ECG monitoring showed stable sinus rhythm without arrhythmia recurrences.

## 3 DISCUSSION

**Figure 3:**
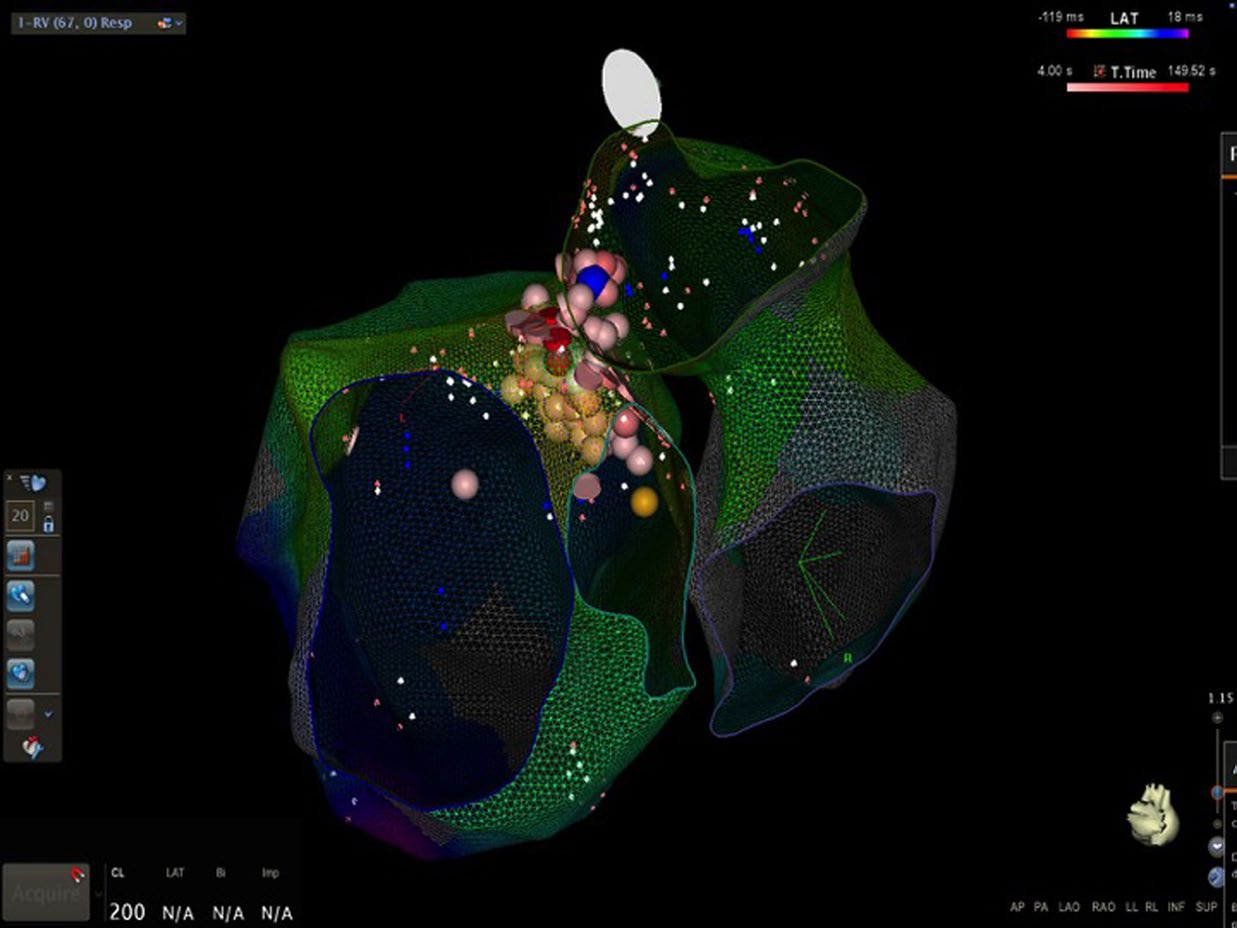
Activation map of the left and right interventricular basal septum. Endocardial breakthrough sites of VA and the sites of RFCA are shown

Our patient presented a nonischemic acute heart failure associated with frequent VAs, without relevant history of heart disease and underlying structural disease. The absence of abnormal local electrogram amplitude during the high‐density substrate mapping suggested that scar zones were not present reinforcing the hypothesis of an idiopathic etiology of ventricular arrhythmia. The 12‐lead ECG analysis showed unique PVC morphology with RBBB pattern, precordial transition in lead V1, and inferior axis suggesting a ventricular outflow tract origin. OT‐VAs are generally idiopathic, occurring in healthy individuals, and more often originate from the RVOT. Four types of LVOT origins have been described in close anatomical proximity: the AMC, the anterior sites around the mitral annulus (MA), the aortic sinus cusps (ASCs), and the epicardium. OT‐VAs may arise from intramural foci and such foci are located between the epicardial fat of the left ventricular summit (LVS) and the AMC or inside the basal septum.^1^ In our case, the LVOT electroanatomical map found the earliest activation site in close proximity to the anterior part of the AMC, just above the interventricular septum (IVS), whereas in the RVOT, the earliest activation site was found in the postero‐septal wall contiguous to the earliest activation site in LVOT (Figure 3). The local presystolic activation time was very similar in both RVOT and LVOT. The presence of equally early sites of activation on opposite sides of IVS and an only transient suppression of the VA ablating from both sides of the septum are suggestive of basal septum intramural foci, as such as the absence of a matching pace‐map at the sites of earliest endocardial ventricular activation.^2^ No ECG features reliably differentiate intramural VAs from the IVS because endocardial breakthrough sites differ between patients.^1^ The management of OT‐VAs originating from basal septum intramural foci is challenging for the technical difficulty to map positioning a catheter in a venous septal perforator branch of the GCV. Moreover, RF catheter ablation may fail to create sufficient lesion when ablating within the venous system for inability to achieve adequate power because of impedance or temperature raise. An alternative approach is to assess the endocardial breakthrough sites on both sides of the septum but sequential or simultaneous unipolar RF ablation from corresponding endocardial sites is required to achieve deep transmural lesions. Intramyocardial infusion‐needle catheter ablation has been used to reach deep intramural foci but it may increase the risk of complications.^3^ In our case, many RF erogations were delivered from both sides of the septum, apparently without reaching the VA focus because PVCs were only transiently suppressed.

In the setting of acute heart failure and frequent VAs may be challenging to determine if VAs are causes or consequences of heart failure. Arrhythmia‐induced cardiomyopathy is a partially or totally reversible disease in which left ventricular dysfunction is induced or mediated by atrial or ventricular arrhythmias.^4^ The detection of this condition requires a high level of suspicion for the presence of persistent arrhythmia with otherwise unexplained cardiomyopathy, and the diagnosis may be evident only after restoration or maintenance of sinus rhythm or after aggressive rate control. The culprit arrhythmia may be supraventricular, for example atrial tachycardia and atrial fibrillation, as such as ventricular with VT or frequent PVCs. The incidence of AIC is 9% to 34% in patients with frequent PVCs.^5^ The primary mechanism of PVC‐induced cardiomyopathy is the abnormal Ca^2+ ^handling associated with impaired excitation‐contraction coupling resulting in impaired systolic and diastolic functions. This dysfunctional calcium metabolism results in prolongation of myocytes action potentials increasing the risk of sudden death even when normal LV size and function are restored.^6^ Additional mechanisms of myocyte remodeling are oxidative stress, myocardial energy depletion, myocardial ischemia, and ventricular dyssinchrony. Alongside cellular changes, remodeling also occurs in the extracellular matrix for the activation of metalloproteinase with loss of extracellular matrix support and LV remodeling and dilatation.^7^ The most important predictor of AIC in patients with frequent PVCs appears to be the daily burden of PVCs with a threshold burden of 10 000 PVCs/day predicting the development of cardiomyopathy.

Our patient was asymptomatic for palpitations and presented without abnormal cardiovascular substrate, with a high PVC burden (>40% of total heartbeats/day), single PVC morphology from ventricular outflow tract, increased body mass index, high PVC coupling interval dispersion. All these features seem to be associated with the development of AIC.^8^ Moreover, it has been showed that patients with AIC have smaller LV end‐diastolic index vs those with other forms of dilated cardiomyopathy and an LV end‐diastolic diameter ≤61 mm is highly predictive of AIC.^9^ In our case, the echocardiogram revealed mild left ventricular dilatation (LVEDD 60 mm), thus raising a high level of suspicion of AIC.

PVC‐mediated AIC is managed suppressing the PVCs through antiarrhythmic therapy or catheter ablation. RFCA is a highly effective therapy with success rates ranging from 70% to 90%. In PVC‐mediated AIC, the complete elimination of high burden PVC, as well as a significant PVC suppression (<5000 PVCs/d), is associated with progressive improvement in heart function and time to recovery varies between few weeks to several months.

After 72 hours from the failed attempt of catheter ablation, PVCs disappeared and LV function progressively improved until normalization during follow‐up. The suppression of PVCs is probably due to the delayed efficacy of RFCA, which has been described after ablation of supraventricular arrhythmias and accessory atrioventricular connections.^10^ Yamada et al^11^ reported delayed efficacy of RFCA for VAs originating from the LV summit. Moreover, delayed efficacy of RFCA has been described after ablation of VAs originating from the LV anterobasal wall and response time to ablation was the only predictor of occurrence of delayed effect.^12^ The postulated mechanism of the delayed effect seems to be the evolution to necrosis and fibrosis of the lesions produced by the RF energy.^13^ RF deliveries produce areas of coagulative necrosis surrounded by an inflammatory cell infiltrate secondary to myocardial damage. This inflammatory response in the peripheral zone may recover completely or produce additional injury leading to posterior fibrosis and lesion extension. In the peripheral area of the ablation sites, the tissue temperature produces cytosolic calcium overload leading to temporary loss of cellular excitability; therefore, the transient suppression of VAs during ablation procedure indicates that RF energy is delivered really close to the focus. In our patient, the fibrotic evolution of inflammatory response and the extension of RF lesions may have affected the breakthrough endocardial sites on the IVS or directly damaged the intramural focus through microvascular ischemia, resulting in delayed suppression of VA.

The awareness of the delayed efficacy of RFCA influences the management of VAs, especially for those arising from LV summit and intramural foci. In these circumstances, RF deliveries on both sides of the IVS, as well as the use of an infusion‐needle catheter, increase the risk of complications. It may be wise to interrupt the procedures waiting for the occurrence of the delayed effect of RFCA. After a failed RFCA, the implant of ICD, as well as a new ablative session, should be procrastinated after a period of follow‐up, for the possible delayed suppression of VAs.

## 4 CONCLUSION

Our patient presents a typical case of AIC secondary to PVCs with recovery of ventricular function after a failed ablation procedure for the occurrence of the delayed efficacy of RFCA. Previous studies have described this phenomenon for VAs originating from the LV summit. This case is, to our knowledge, the first description of RF delayed efficacy in VAs arising from intramural focus in the basal IVS and demonstrates that waiting for delayed efficacy may be a reasonable choice in order to reduce the risk of complications associated with more aggressive approaches.

## CONFLICT OF INTEREST

The authors declare no conflict of interest.

## AUTHOR CONTRIBUTION

MVM: involved in the conception and drafting the manuscript. MCG, AP, and CL: performed the electrophysiological study and the radiofrequency catheter ablation procedure. FF: critically reviewed the manuscript.
